# Oral Valacyclovir Treatment of Herpes Zoster Ophthalmicus-Induced Optic Neuritis

**DOI:** 10.7759/cureus.17033

**Published:** 2021-08-09

**Authors:** Christina M Hunt, Hannah M Gregory, William Gannon

**Affiliations:** 1 Department of Research, Alabama College of Osteopathic Medicine, Dothan, USA; 2 Ophthalmology, Eye Surgical Associates, Dothan, USA

**Keywords:** herpes zoster ophthalmicus, valacyclovir, acyclovir, optic neuritis, optic nerve

## Abstract

Herpes zoster ophthalmicus (HZO) rarely manifests with complications of the optic nerve. The traditional standard of care for sight-threatening HZO infection involves intravenous hospital administration of the antiviral medication acyclovir. This case report entails an HZO complication invading the optic nerve, effectively treated by oral administration of the antiviral medication valacyclovir in an immunocompetent patient. Intravenous administration of antiviral medication may be undesirable for some patients with HZO due to comparative cost, stronger associations to nephrotoxicity, increased dosing frequency, and the need for hospitalization. Oral antiviral tablets have an efficacious route of administration to be considered over intravenous hospital administration when devising treatment for HZO with the rare complication of optic neuritis in immunocompetent patients.

## Introduction

Herpes zoster virus results from the reactivation of a latent varicella-zoster virus [1]. Herpes zoster infects 20-30% of the population and presents with a painful prodrome of symptoms, including eye pain, ocular changes, or skin rash [1]. Ten to twenty percent of patients with herpes zoster will develop herpes zoster ophthalmicus (HZO) [1]. HZO has rarely been shown to involve optic nerve complications. There is no agreed-upon consensus for the medical management of HZO, though oral or intravenous administration of the antiviral agent acyclovir or valacyclovir is commonly used as first-line drugs [2]. This case report depicts a complication of HZO involving the optic nerve with papilledema and hemorrhage effectively managed by oral tablet administration of the antiviral medication valacyclovir and adjuncts.

## Case presentation

A 54-year-old caucasian male presented to the emergency department for left eye pain. The patient complained of blurred vision, burning sensation of the eye, light sensitivity, redness, tearing, and eye pain that had gradually worsened. The patient had no significant past medical, family, or social history. He was found to have a shingles outbreak on the left side of his face and was subsequently referred to an ophthalmology clinic for further evaluation.

At the clinic, physical examination revealed a vesicular rash following a dermatomal pattern on the scalp, forehead, and nose on the left side. The right eye demonstrated 20/20 vision and the left was 20/25. On pupillary examination, there was initially no afferent pupillary defect (APD). Upon anterior segment examination, the intraocular pressure was recorded 10 mmHg in the right eye and 14 mmHg in the left eye. On the left eye, the patient had conjunctival injection and chemosis but no evidence of corneal dendrites or pseudodendrites. However, his anterior chamber did have 1-2+ cellular reactions along with some keratic precipitates. On a posterior segment examination of the left eye, his optic nerve was found to be sharp, pink, and healthy without evidence of edema or hemorrhage.

The patient was prescribed one difluprednate 0.05% eye drop four times per day and oral valaciclovir 1,000 mg three times per day and told to follow up in one week unless symptoms worsened. The patient was compliant with all treatment instructions and at the follow-up visit one week from the initial presentation, the chemosis and erythema in the left eye had resolved. However, the patient complained of slightly worse vision. He was found to have an APD in the left eye.

**Figure 1:**
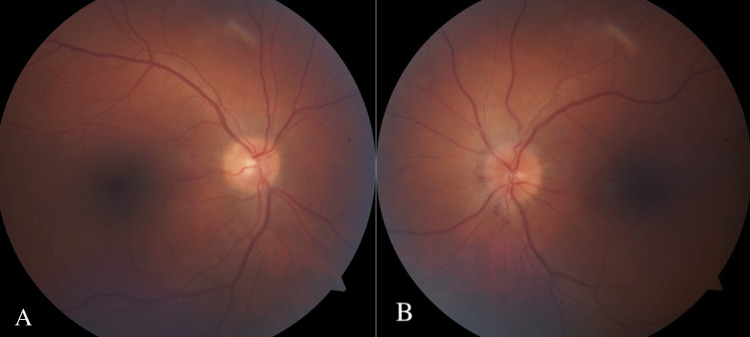
Fundoscopic imaging from initial examination of the right eye (A) and the left eye (B) showing left optic disc papilledema and hemorrhage in the left eye.

**Figure 2:**
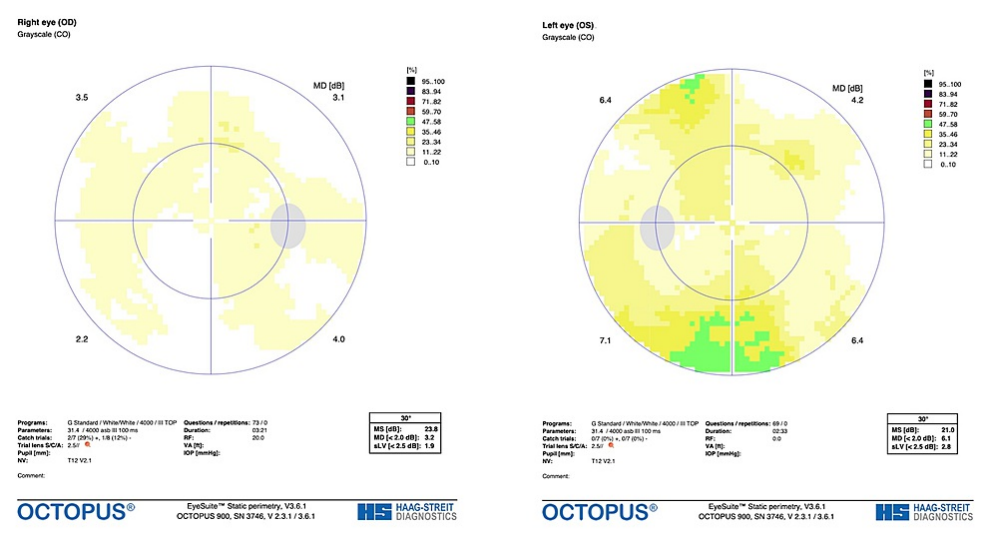
Visual field grayscale imaging of the left eye (left) and right eye (right) from initial examination.

**Figure 3:**
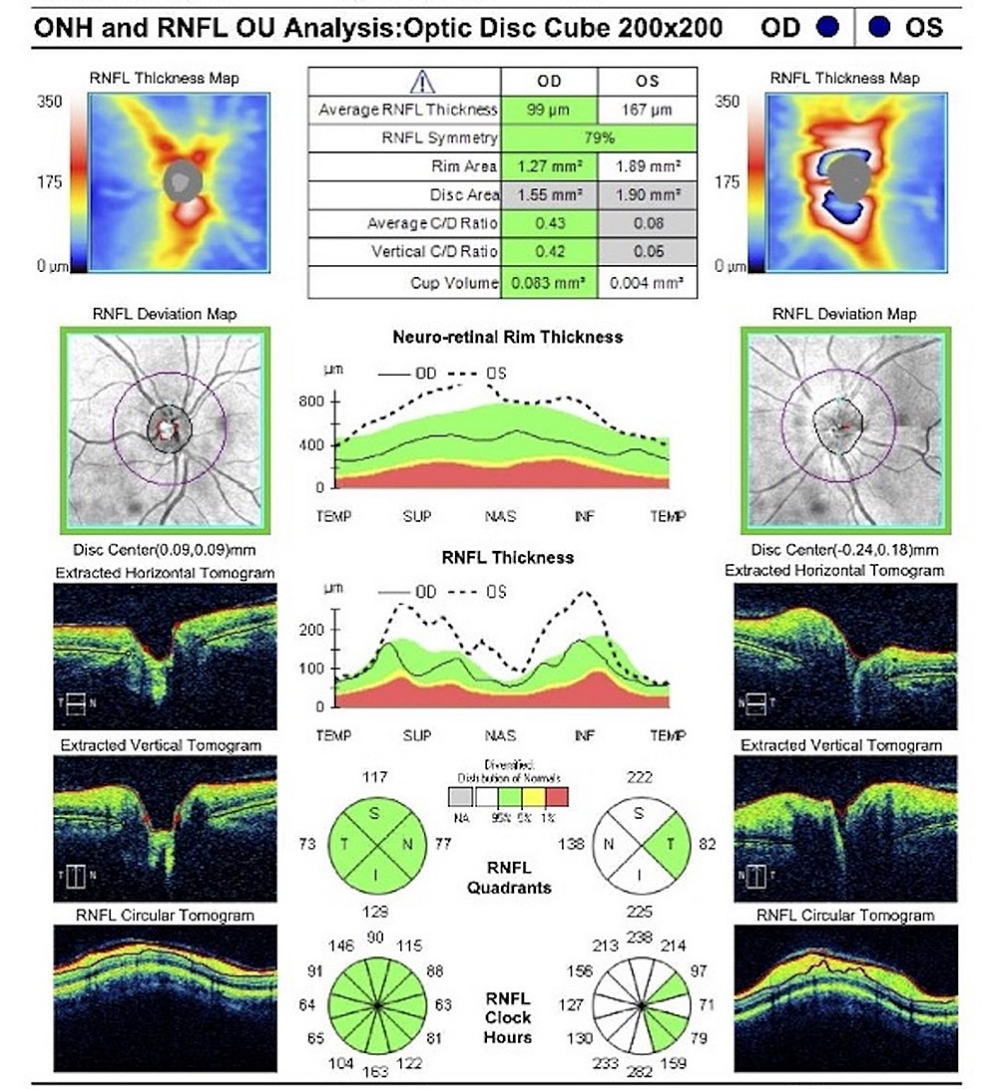
Optic nerve head and retinal nerve fiber layer analysis report.

At the time of follow-up, the right eye had no significant fundoscopic findings (Figure 1). Fundoscopic examination revealed papilledema and hemorrhage of the left nasal and inferior part of the left optic nerve (Figure 1). Visual field testing was also performed (Figure 2) and an optic nerve head (ONH) and retinal nerve fiber layer (RNFL) analysis were generated (Figure 3). Visual field testing showed a full field in the right eye and depression of the visual field in the left eye. Optic nerve testing using optical coherence tomography showed edema of the optic nerve head. Given the new optic nerve involvement and findings, the patient was diagnosed with HZO with optic neuritis.

**Figure 4:**
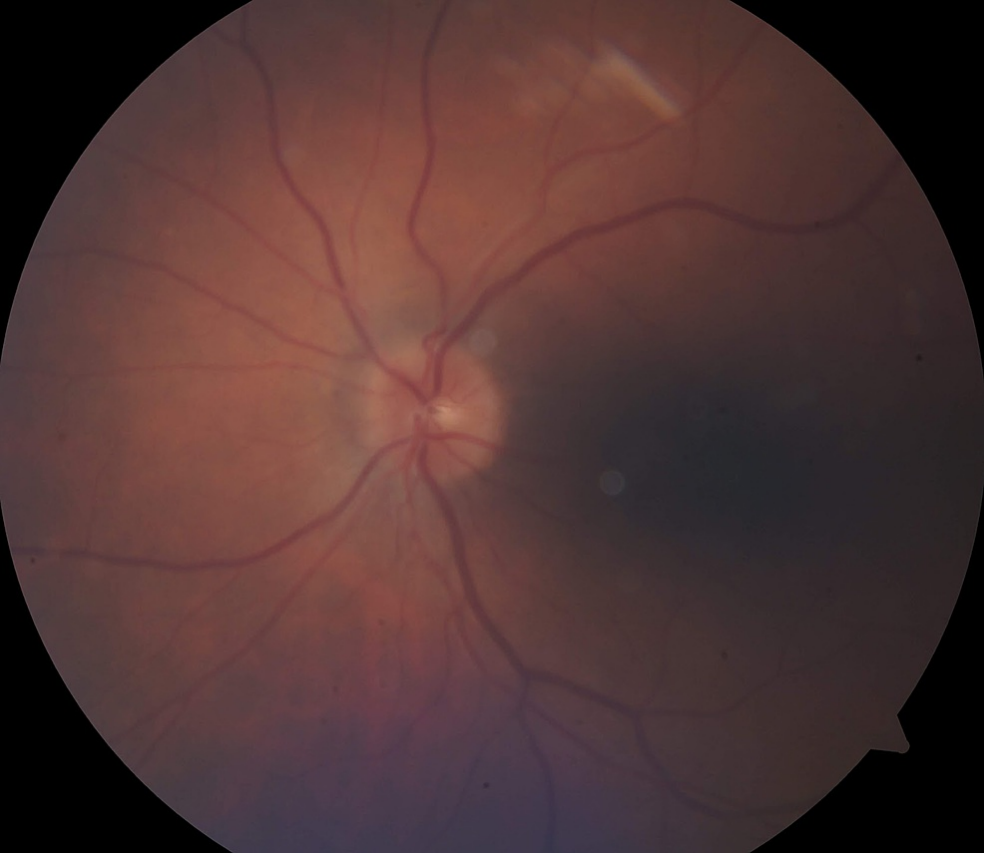
Fundoscopic image from the two-week follow-up visit showing the resolved optic disc edema and hemorrhage of the left eye.

**Figure 5:**
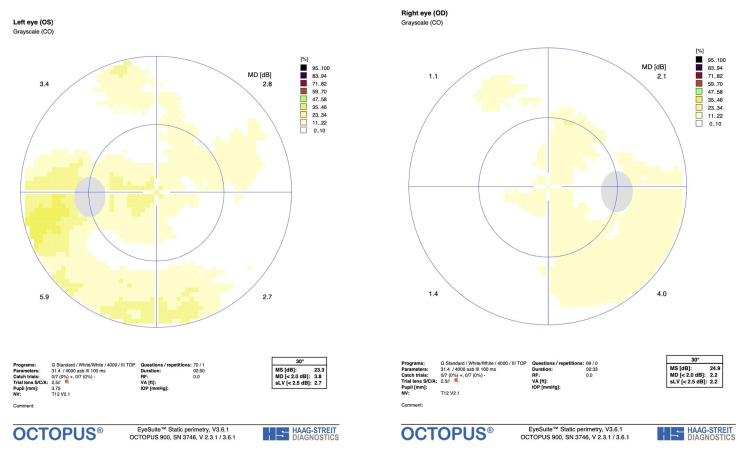
Visual field imaging of left eye upon two-week follow-up visit showing optic disc resolution (left) and the right eye comparison (right).

The decision was made to continue the patient on oral valacyclovir at 1 g three times daily and instructions were given to follow up in one week or sooner if vision changes presented. Hospitalization for intravenous antiviral medication was considered, but given the patient’s good health otherwise and noted compliance, it was decided to continue oral medication as long as the patient’s ocular issues did not worsen. The patient returned one week later with vision consistent with his baseline and APD present. Fundoscopic examination (Figure 4) and visual field testing (Figure 5) were performed and showed resolved disc hemorrhage and improved visual field. The iritis resolved and only very subtle optic disc edema was noted. Oral valacyclovir was continued at 1,000 mg three times per day and oral prednisone at 10 mg three times per day was added. At seven weeks from the initial presentation, the patient showed complete resolution of optic nerve symptoms. At a follow-up appointment five months after the patient stopped the valacyclovir regimen, there was no sign of HZO virus upon examination.

## Discussion

HZO occurs when the latent varicella-zoster virus becomes reactivated in the ophthalmic (V1) division of the trigeminal nerve [3]. Risk factors for reactivation of HZO are age over 50-years-old, immunosuppression, chronic disease, acute illness, and physical and emotional stressors, including trauma [4]. HZO is a manifestation of the herpes zoster virus, present in roughly 20% of all herpes zoster cases, that affects the periorbital region and the trigeminal nerve (V1) dermatome distribution [2,3]. Without adequate and effective treatment, HZO can persist with associated vision loss, glaucoma, central retinal artery occlusion, dendritic keratitis, corneal anesthesia, secondary infection, stromal neovascularization, corneal opacity, chronic granulomatous uveitis, iris atrophy, scleritis, and acute retinal necrosis [2,3,5]. However, the most debilitating complication of HZO is postherpetic neuralgia (PHN), a neuropathic pain syndrome that persists after the zoster rash has resolved [1]. In some cases, HZO has even shown an association with increased risks of hemorrhagic or ischemic complications including subsequent transient ischemic attacks, strokes, and myocardial infarctions due to inflammation and vascular remodeling, and thickening caused by varicella-zoster virus infection [6]. Patients with documented HZO exposure have a 1.3 to 4-fold increased risk of having a cerebrovascular event following the exposure, with the risk being even higher among younger patients [6]. Current preventive methods for the care of HZO include a live, attenuated vaccine for immunocompetent patients or a recombinant version for immunocompromised patients [2]. Both vaccines can reduce the risk of viral reactivation, however, the vaccines only last for about eight years and are less effective in patients over 70 years [2].

Optic neuritis is a rare complication of HZO that was presented in this case report. Optic neuritis is a severe and acute ophthalmic condition that can result in vision loss if not treated effectively [7]. This complication has been reported in 1.9% of HZO-affected eyes [8]. The manifestation of optic neuritis is due to inflammation in the central nervous system, resulting in edema and inflammation of the optic nerve. Optic neuritis is typically seen with multiple sclerosis but it can accompany HZO in rare cases. The patient, in this case, was found to have optic neuritis manifesting as vision loss, APD, and edema of the optic nerve head. Treatment of optic neuritis typically requires corticosteroids [7], which were prescribed to the patient in this case along with an antiviral agent.

The current treatment regimen of HZO often includes oral or intravenous administration of the antiviral agent acyclovir as a first-line drug [2]. Treatment with antiviral therapy reduces the risk of chronic ocular complications by 20% to 30% [2]. Antiviral therapy does not prevent PHN from developing, but reduces the duration of the PHN pain by about 50% and helps prevent severe ocular damage [2]. In the case of this patient, the antiviral agent valacyclovir was initially prescribed at 1,000 mg, three times per day, via oral administration. At the initial one-week follow-up appointment, the decision was made to continue the patient on the oral valacyclovir treatment regimen due to the resolution of chemosis in the left eye and erythema. The patient continued this course of antiviral therapy for seven weeks until the optic nerve of the left eye had resolved and there were no signs of optic disc hemorrhage, edema, or visual field defects. At a follow-up appointment five months after the patient stopped their oral valacyclovir regimen, there was no sign of HZO virus upon examination of the eye and the optic nerve had no abnormality noted. 

Oral valacyclovir therapy was chosen in this case as the patient was immunocompetent with no other comorbidities (i.e., renal impairment) that would have complicated the treatment regimen or indicated the need for disseminated intravenous therapy. Hospital admission may be recommended for patients with HZO as intravenous administration of an antiviral agent may be needed for treatment. Intravenous antiviral agents like acyclovir have increased bioavailability when compared to oral formulations, which is why they are recommended for HZO treatment in immunocompromised patients or patients with the sight-threatening disease [9,10].

Valacyclovir and acyclovir are the most commonly used antiviral medications to treat HZO [10]. Unlike acyclovir which can be administered both intravenously and orally, valacyclovir is only administered orally. Oral valacyclovir gives a three to fivefold increase in acyclovir bioavailability as it is rapidly converted to acyclovir upon administration in vivo [11]. The decreased bioavailability of acyclovir means that more frequent dosing is required for adequate treatment of herpes zoster. A higher dose (1,000 mg) of valacyclovir can be effectively administered three times per day while a lower dose (800 mg) of acyclovir may be administered up to five times per day for a standard treatment regimen of herpes zoster in adults [9,12]. The increased dosing frequency of acyclovir may lead to lower patient compliance [13]. The more convenient dosing schedule of valacyclovir and the elimination of the need for hospitalization due to its oral administration route are two important factors to consider when electing an antiviral agent for the treatment of HZO.

Another factor to consider when deciding which antiviral agent to prescribe for the treatment of HZO is the presence of renal impairment. IV agents may be more likely to increase the risk of nephrotoxicity due to higher peak levels in vivo and subsequent precipitation of crystals in the renal tubules [12]. Intravenous acyclovir administration is associated with acute kidney injury and acute renal failure due to intra-tubular crystal precipitation, rapid excretion in the urine, and relatively low solubility [9,14,15]. The cost of treatment to the patient may also be considered when deciding between antiviral agents for the treatment of HZO. The use of valacyclovir prophylactically has been associated with increased cost savings and feasibility of use [16].

One limitation of the use of oral valacyclovir is that it is not the recommended first-line therapy of choice in immunocompromised patients. Acyclovir is typically the drug of choice for the treatment of herpes zoster in immunocompromised patients due to its ability to achieve higher peak levels in vivo with intravenous administration [17]. However, valacyclovir has been demonstrated to show equal efficacy to acyclovir when used in immunocompromised patient populations ages 18 years and older [18]. Therefore, the use of oral valacyclovir in immunocompromised patients as a first-line agent should be further examined.

Valacyclovir is an efficacious agent of choice as it eliminates the need for hospitalization and intravenous administration, decreases the risk of developing renal impairment, and increases cost savings to the patient, while successfully treating HZO with complex optic nerve involvement. Oral valacyclovir should be considered instead of an intravenous antiviral agent for the treatment of HZO in immunocompetent patients who do not require hospitalization for treatment of disseminated infection. The case of this 54-year-old patient who was successfully treated with oral valacyclovir instead of an intravenous agent demonstrates the usefulness of valacyclovir in HZO therapy with the complication of optic neuritis. Future research may further explore the effectiveness of oral valacyclovir in treating other rare complications of HZO.

## Conclusions

HZO rarely manifests with complications of the optic nerve including papilledema and hemorrhage. Oral valacyclovir was found to be an effective treatment choice for treating optic nerve complications seen in an immunocompetent patient with HZO. The traditional treatment regimen for HZO has been hospital administration of intravenous antiviral medication, however, this may not be the most appropriate therapy for otherwise healthy patients considering cost, time, and convenience. Oral route administration over intravenous hospital administration should be considered by providers when devising a treatment regimen for patients with HZO.
